# 

**Published:** 1906-04-07

**Authors:** 


SPECIAL NUMBER. SPECIAL NUMBER.

### THE HOSPITAL

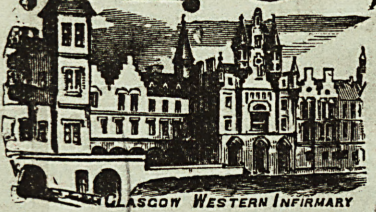

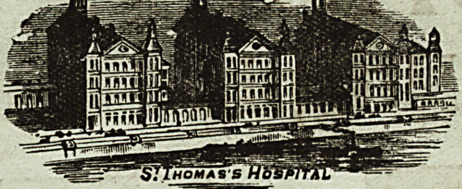

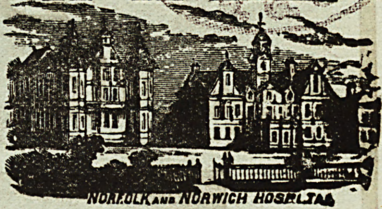

No. 1,019. Vol. XL. [Registered as a Newspaper.] SATURDAY, APRIL 7TH, 1906. [Subscription Terms see p. 26.] PRICE THREEPENCE

